# Sulphate of Quinine Not Absorbed When Applied Endemically

**Published:** 1846-04

**Authors:** 


*Sulphate of Quinine not absorbed when applied Endemically.* By M. Martin-Solon.—Many medicines, when applied to the skin, either whole or deprived of its cuticle, act energetically on the economy, and may be detected in the secretions, thus showing they have been absorbed. Sulphate of quinine, when given internally in the dose of one grain, may easily be detected in the urine by means of the ordinary tests, as iodide of potassium, &c. Martin Solon, however, has made many experiments on twenty individuals affected with various maladies, relative to this medicine being absorbed when applied to the skin, and in no case has he succeeded in detecting the slightest traces of the medicine in the urine. The sulphate of quinine was applied by friction to the sound skin, and to that denuded of cuticle, in baths, and by means of ointments. The effect was null in all.—*New Orleans Med. and Surg. Journal, from Bulletin de Therapeutique.*

